# Early decreased TLR2 expression on monocytes is associated with their reduced phagocytic activity and impaired maturation in a porcine polytrauma model

**DOI:** 10.1371/journal.pone.0187404

**Published:** 2017-11-10

**Authors:** Lukas Schimunek, Rafael Serve, Michel P. J. Teuben, Philipp Störmann, Birgit Auner, Mathias Woschek, Roman Pfeifer, Klemens Horst, Tim-P. Simon, Miriam Kalbitz, Ramona Sturm, Hans-C. Pape, Frank Hildebrand, Ingo Marzi, Borna Relja

**Affiliations:** 1 Department of Trauma-, Hand- and Reconstructive Surgery, University Hospital Frankfurt, Goethe University, Frankfurt/Main, Germany; 2 Department of Orthopaedic Trauma Surgery, University Hospital Zurich, University of Zurich, Zurich, Switzerland; 3 Department of Orthopaedic Trauma, RWTH Aachen University, Aachen, Germany; 4 Department of Intensive Care and Intermediate Care, RWTH Aachen University, Aachen, Germany; 5 Department of Orthopedic Trauma, Hand, Plastic, and Reconstructive Surgery, University of Ulm, Ulm, Germany; Universitatsklinikum Freiburg, GERMANY

## Abstract

In their post-traumatic course, trauma patients suffering from multiple injuries have a high risk for immune dysregulation, which may contribute to post-injury complications and late mortality. Monocytes as specific effector cells of the innate immunity play a crucial role in inflammation. Using their Pattern Recognition Receptors (PRRs), notably Toll-Like Receptors (TLR), the monocytes recognize pathogens and/or pathogen-associated molecular patterns (PAMPs) and organize their clearance. TLR2 is the major receptor for particles of gram-positive bacteria, and initiates their phagocytosis. Here, we investigated the phagocytizing capability of monocytes in a long-term porcine severe trauma model (polytrauma, PT) with regard to their TLR2 expression. Polytrauma consisted of femur fracture, unilateral lung contusion, liver laceration, hemorrhagic shock with subsequent resuscitation and surgical fracture fixation. After induction of PT, peripheral blood was withdrawn before (-1 h) and directly after trauma (0 h), as well as 3.5 h, 5.5 h, 24 h and 72 h later. CD14^+^ monocytes were identified and the expression levels of H(S)LA-DR and TLR2 were investigated by flow cytometry. Additionally, the phagocytizing activity of monocytes by applying *S*. *aureus* particles labelled with pHrodo fluorescent reagent was also assessed by flow cytometry. Furthermore, blood samples from 10 healthy pigs were exposed to a TLR2-neutralizing antibody and subsequently *to S*. *aureus* particles. Using flow cytometry, phagocytizing activity was determined. P below 0.05 was considered significant. The number of CD14^+^ monocytes of all circulating leukocytes remained constant during the observational time period, while the percentage of CD14^+^H(S)LA-DR^+^ monocytes significantly decreased directly, 3.5 h and 5.5 h after trauma. The percentage of TLR2^+^ expressing cells out of all monocytes significantly decreased directly, 3.5 h and 5.5 h after trauma. The percentage of phagocytizing monocytes decreased immediately and remained lower during the first 3.5 h after trauma, but increased after 24 h. Antagonizing TLR2 significantly decreased the phagocytizing activity of monocytes. Both, decreased percentage of activated as well as TLR2 expressing monocytes persisted as long as the reduced phagocytosis was observed. Moreover, neutralizing TLR2 led to a reduced capability of phagocytosis as well. Therefore, we assume that reduced TLR2 expression may be responsible for the decreased phagocytizing capacity of circulating monocytes in the early post-traumatic phase.

## Introduction

Traumatically induced tissue damage leads to an inflammatory response. Inflammation itself is not detrimental but rather necessary for the resolution of injury and the healing progress. This complex process integrates and coordinates cytokines, chemokines and immune cells to deal with the damage.[1, 2] One attempt to characterize this inflammatory reaction was the concept of a trauma-induced hyper-inflammatory systemic inflammatory response syndrome (SIRS) and the counterbalancing hypo-inflammatory state (compensatory anti-inflammatory response syndrome, CARS) in the later clinical course.[1, 3] The balance between pro- and anti-inflammatory components, the SIRS-CARS paradigm, was assumed to be crucial for a successful recovery and a positive outcome.[1, 4, 5] However, the classification terms of SIRS and CARS are more than 20 years old, and are only of limited usefulness to describe the patient’s current immune status because they do not always correlate well with immunofunctional parameters. Moreover, the injured tissue releases a large number of soluble factors that act on the endocrine, lymphoid and haematopoietic organs as well.[2]

Monocytes play a crucial role in the early immune response after trauma and infection. On one hand they constitute a cellular link between the innate and the adaptive immune system in case of infection, and on the other hand, are capable of recognizing pathogens or pathogen-associated molecular patterns (PAMPs) directed by their pattern recognition receptors (PRRs), and subsequently inactivate invading pathogens by phagocytosis.[6–8] The impact of trauma onto the function of monocytes is still not clear and can be conflicting. Anupamaa Seshadri *et al*. (2017) found significantly increased levels of circulating monocytes in trauma patients in the first five days after trauma compared to healthy volunteers.[9] Conversely, they found a significant depression of monocytic cytokine production (TNF-α, IL-1β) as well as significantly impaired expression of MHC-II molecules on the surface, while the phagocytic capability was not affected over the 5 days post-trauma.[9] Heftrig *et al*. (2017) reported different results for the circulating levels of monocytes, while a constant depression of MHC-II expression as well as an impaired production of IL-1β over the time course of ten days after trauma compared to healthy volunteers was observed.[10]

An important subset of PRRs are Toll-like Receptors (TLRs), of which there are 10 known types in humans.[11–13] TLR2 is the major receptor for bacterial lipoproteins, lipopeptides and lipoteichoic acid (LTA), which are common for gram-positive bacteria.[14] *S*. *aureus* is the most common gram-positive bacterium, which causes nosocomial infections like pneumonia and sepsis, and is therefore highly associated with increased morbidity and mortality in chronically ill patients.[15–17] Furthermore, *S*. *aureus* is feared for its potential to infect wounds and enter the bloodstream after trauma or surgery.[18, 19] Phagocytosis seems to be initiated by the activation of PRRs, and in particular TLRs.[20] Several studies show an impaired phagocytic capability of monocytes in the post-traumatic course as described below.[21, 22] The data is inconsistent regarding TLR2 expression on monocytes after trauma. Perez-Barcena *et al*. (2010) have shown increased levels of TLR2 on monocytes in trauma patients compared to healthy volunteers over a time course of 14 days.[21] They further have shown a significantly decreased expression of TLR2 in patients who developed any infection compared to those without infections.[21] Furthermore, they have shown an impaired phagocytic activity of monocytes during the 14 days in trauma patients compared to healthy volunteers.[21] In contrast to those findings, Adib-Conquy *et al*. (2003) reported a constant TLR2 expression on monocytes in trauma patients at admission compared to healthy volunteers.[23] Other studies have shown an impaired expression of TLR2 in trauma patients during the first 48 hours or over a time course of 10 days after trauma compared to healthy volunteers.[10, 24]

Expression of the antigen presenting human leukocyte antigen (HLA) molecules, the cell surface proteins human major histocompatibility complex MHC-II, has been known for a long time and is well described on human monocytes from healthy volunteers.[25, 26] However, early after hemorrhagic shock or severe abdominal surgery, an impaired MHC-II expression on macrophages and specifically on monocytes has been reported.[27, 28] With regard to trauma, a decreased MHC-II expression in human monocytes after trauma has been reported.[10, 29] Nonetheless, the expression profile of H(S)LAs, the porcine MHC molecules, still remains not fully discovered, and even less is known about their behavior after trauma. MHC-I and MHC-II molecules were first observed on monocytes in pigs by Chamorro *et al*.[30] Raymond *et al*. (2005) also described the expression of MHC-II on porcine monocytes, showing an increase after adding both lipopolysaccharide (LPS) or lipoteichoic acid (LTA).[31]

To summarize, there are alterations in TLR2 and MHC-II expression, as well as modulations in phagocytizing behavior of monocytes after severe trauma, and the discrepancy between the results in previous studies is tremendous. And still, mechanisms are not discovered yet, and even less is known with regard to the experimental polytrauma model in a large animal. The varied findings in the mentioned studies above could be due to the uncontrollable nature of trauma and its complications. Several studies only ackowledged one time point in the clinical course (e.g. admission or in the first 48 hours after admission). Furthermore, there are very few studies that combine phenotyping of immune cells with their physiological function after trauma. Therefore, in our experiment, the expression of MHC-II (SLA-DR) and TLR2 on circulating monocytes were measured in a time course of 72 hours after severe trauma in a controlled porcine long term polytrauma model. In parallel, the phagocytizing capacity of the monocytes was evaluated as one of their physiological function. Furthermore, the direct association of TLR2 with phagocytosis was analyzed. In this prospective study, we hypothesized we would observe similar results in the individual parts of the study as in previous human trauma studies with two goals: first to establish the porcine polytrauma model as a way to further investigate patients’ post-traumatic physiology immune response, and second to associate our individual parts of the study to get more knowledge about mechanistics behind post-traumatic immune (dys-) regulation.

## Material and methods

### Ethics

The experiments were authorized by a responsible government authority ("Landesamt für Natur, Umwelt und Verbraucherschutz": LANUV-NRW, Germany: AZ TV-Nr.: 84–02.04.2014.A265) and performed in compliance with the federal German law with regards to the protection of animals, Institutional Guidelines and the criteria in “Guide for the Care and Use of Laboratory Animals” (Eighth Edition The National Academies Press, 2011).[32] In our study, we handled the animals consistently in accordance with the ARRIVE guidelines.[33] Animal experiments were performed at the Institute for Laboratory Animal Science & Experimental Surgery, RWTH Aachen University, Germany.

### Animals

A total of twelve male German landrace pigs (*Sus scrofa*; 3 months old, 30 ± 5 kg) from a disease-free barrier breeding facility were included in this study. Placed in air conditioned rooms, all animals were examined by a veterinarian and allowed to acclimatize to their environment for at least 7 days prior to the experiments. The night before the experiments, the pigs were fasted but had a free access to water.

This study presents partial results obtained from a large animal porcine multiple trauma model. The model has been previously described in detail by Horst et al.[34]

### Anesthesia and preparation

Animals were pre-medicated with an intramuscular (IM) application of Azaperone (Stresni^TM^, Janssen, Germany) in a dose of 4 mg/kg. Anesthesia was induced with an intravenous injection of Propofol (3 mg/kg) followed by orotracheal intubation (7.5 ch tube, Hi-Lo Lanz^TM^). During the study period over 72 h, anesthesia and analgesia was continuously maintained with intravenous (IV) injection of Propofol and Sufentanil at a sufficient level to prevent any periods of pain or consciousness. The animals were not awakened at any time point after the induction of polytrauma. The animals were ventilated on volume control mode (Draeger, Evita, Lübeck, Germany) with room air at a tidal volume setting of 6–8 ml/kg, positive end expiratory pressure (PEEP) of 8 mmHg (plateau pressure < 28 mmHg), and pCO_2_ of 35–45 mm Hg as previously described.[34] Catheters were aseptically inserted in multiple locations: the external jugular vein for administration of fluids, anesthesia and continuous monitoring of central venous pressure (CVP, central venous catheter 4-Lumen Catheter, 8.5 Fr., ArrowCatheter, Teleflex Medical, Germany), the right femoral vein to induce hemorrhage (3-Lumen hemodialysis, 12.0 Fr., ArrowCatheter, Teleflex Medical, Germany) and into the femoral artery for continuous blood pressure monitoring (4.0 Fr. arterial line catheter, Vygon, Germany). A urinary catheter was also inserted in the bladder (12.0 Fr, Cystofix, Braun, Melsungen, Germany). Crystalloid fluid (Sterofundin ISO^®^) was used for continuous fluid management (2 ml kg/BW/h). The baseline measurements were acquired after instrumentation and calibration, prior to starting experimentation.

### Induction of polytrauma

The polytrauma was induced as previously described.[34] In brief, antibiotic prophylaxis (Ceftriaxon® 2 g) was administered before surgery and after every 24 h until sacrifice. Prior to initial trauma induction, the fraction of inspired O_2_ (FiO_2_) was set at 0.21 and the fluid administration was reduced to 10 ml/h. At this phase, the animals were allowed to descend into a hypothermic state following hemorrhagic shock period mimicking the pre-clinical scenario. The animal was positioned on the right side and a femur fracture was induced with a bolt shot on the right hind leg (Blitz-Kerner, turbocut JOBB GmbH, Germany, 9x17, Dynamit Nobel AG, Troisdorf, Germany). After being placed back in the dorsal position, blunt thoracic trauma with a bolt shot on the right dorsal lower thorax was induced. Finally, a midline-laparotomy and uncontrolled bleeding for 30 seconds after crosswise incision of the caudal liver lobe (4.5 x 4.5 cm) was induced. Using sterile gauze-compresses the liver was packed. Pressure-controlled haemorrhagic shock using exsanguination from right femoral artery was performed until a mean arterial blood pressure (MAP) of 40 ± 5 mm Hg was reached and maintained for 90 minutes.

Resuscitation started immediately after hemorrhagic shock by adjusting FiO_2_ to baseline values, and re-infusing with previously withdrawn blood and additional fluids (Sterofundin ISO^®^; 2 ml kg/BW/h). Rewarming was performed using forced-air warming systems until normothermia was reached (38.7–39.8°C).

After experimentation, clinical treatment of the open femur fracture was performed according to established trauma guidelines. The intensive care and complications management followed the standardized clinical protocols according to the latest recommendations of the European Resuscitation Council and Advanced Trauma Life Support (ATLS).[35, 36] After the observational period the animals were euthanized by using potassium chloride until cardiac arrest.

### Blood sampling

Blood samples were withdrawn directly after surgery in ethylenediaminetetraacetic acid (EDTA) tubes (Sarstedt, Nürmbrecht, Germany) before trauma induction as control at 3.5 h, 5.5 h, 24 h and 72 h after trauma. The samples were kept at room temperature until subsequent analyses.

### *Ex vivo in vitro* whole blood stimulation for phagocytosis assay

Blood samples (40 μl) were transferred into polystyrene FACS tubes (BD Pharmingen^TM^) and incubated with 40 μl of pH Rodo red (pHrodo® Red *S*. *aureus* BioParticles® Conjugate for Phagocytosis, ThermoFisher, Germany) for 1 h at 37°C, 5% CO_2_ according to the manufacturer’s instructions. A negative control without *S*. *aureus* Red BioParticles Conjugate was included. Afterwards, 1 ml of FACS lysing solution (FACS Lysing Solution, 1:10, BD Pharmingen^TM^, Heidelberg, Germany) was added, followed by another incubation step (10 minutes) for red blood cells lysis. Thereafter, 2 ml of phosphate buffered saline (PBS) were added and the samples were centrifuged at 800G for 8 minutes at room temperature. Subsequently, the samples were washed with 3 ml of PBS with supplements (0.5% bovine serum albumin (BSA), FACS buffer) and centrifuged again at 800G for 8 minutes. The pellet was resuspended in 400 μl FACS buffer. The measurement was performed by flow cytometry using *BD FACS Canto*^*TM*^ and *FACS DIVA*^*TM*^ software (FACSCanto II, BD Biosciences). The monocyte population was discriminated by forward and sideward scatter. 300,000 monocytes were measured in each sample. The phagocytizing activity of monocytes was quantified as a percentage of cell population gated.

### *Ex vivo in vitro* whole blood cell surface receptor analysis

Blood samples (100 μl) were incubated for 30 minutes at room temperature in darkness with mouse anti-human CD14 PE (Clone TÜK4, BD Bioscience), mouse anti-human HLA-DR PerCP-Cy5.5 (Clone HL-38, Novus Biologicals), or polyclonal rabbit anti-human TLR2 Alexa Fluor 700 (Bioss Antibodies, 5 μl). Control samples were incubated with suggested isotype controls for the settings. After incubation, 3 ml of FACS lysing solution (BD Pharmingen^TM^) were added and incubation for 10 minutes at room temperature in darkness followed. Subsequently, the samples were centrifuged at 800G for 5 minutes at room temperature. The pellet was resuspended in 500 μl of FACS buffer. The samples were measured by flow cytometry with *BD FACS Canto*^*TM*^ using *FACS DIVA*^*TM*^ software (BD Biosciences). The monocytes were discriminated by gating CD14^+^ cells. 300,000 monocytes were measured at least from each sample. Unstimulated samples were measured as control.

### TLR2 neutralization followed by phagocytosis assay

Blood samples (EDTA tubes, Sarstedt) from ten male Pietrain pigs (*Sus scrofa*, 6 months old, 100 ± 5 kg) from a disease-free barrier breeding facility Bundes Hybrid Zucht Programm (BHZP) were drawn immediately after slaughtering for mechanistic studies. 50 μl were transferred into polystyrene FACS tubes (BD Pharmingen^TM^). The samples were kept at room temperature and incubated for one hour at room temperature in darkness with the rat IgG polyclonal neutralizing human TLR2 (Invivogen) (20 μg/ml). Control samples were incubated for one hour at room temperature in darkness with the normal rat IgG control PAb antibody (Invivogen) (20 μg/ml) or without antibodies (ctrl). In the following, 40 μl of each sample were incubated with pH Rodo red bioparticles (pHrodo® Red *S*. *aureus* BioParticles® Conjugate for Phagocytosis, ThermoFisher, Germany) according to the manufacturer’s instructions to determine the phagocytizing capacity of cells (see protocol above). The monocyte population was discriminated by forward and sideward scatter. 300,000 monocytes were measured in each sample. The phagocytizing activity of monocytes was quantified as a percentage of cell population gated.

### Statistical analysis

All statistical analyses were performed employing GraphPad Prism 6 (Graphpad Software, Inc., San Diego, CA). D’Agostino-Pearson normality test was applied to test the normality of data. To compare the differences between the groups, the matched-pair statistical analysis was performed by using repeated measures ANOVA (Friedman test) with a Dunn`s post-hoc test. A p value of less than 0.05 was considered significant. Data are given as mean ± standard error of the mean (sem).

## Results

### Ratio of CD14^+^ monocytes to leukocytes

**Fig 1:**
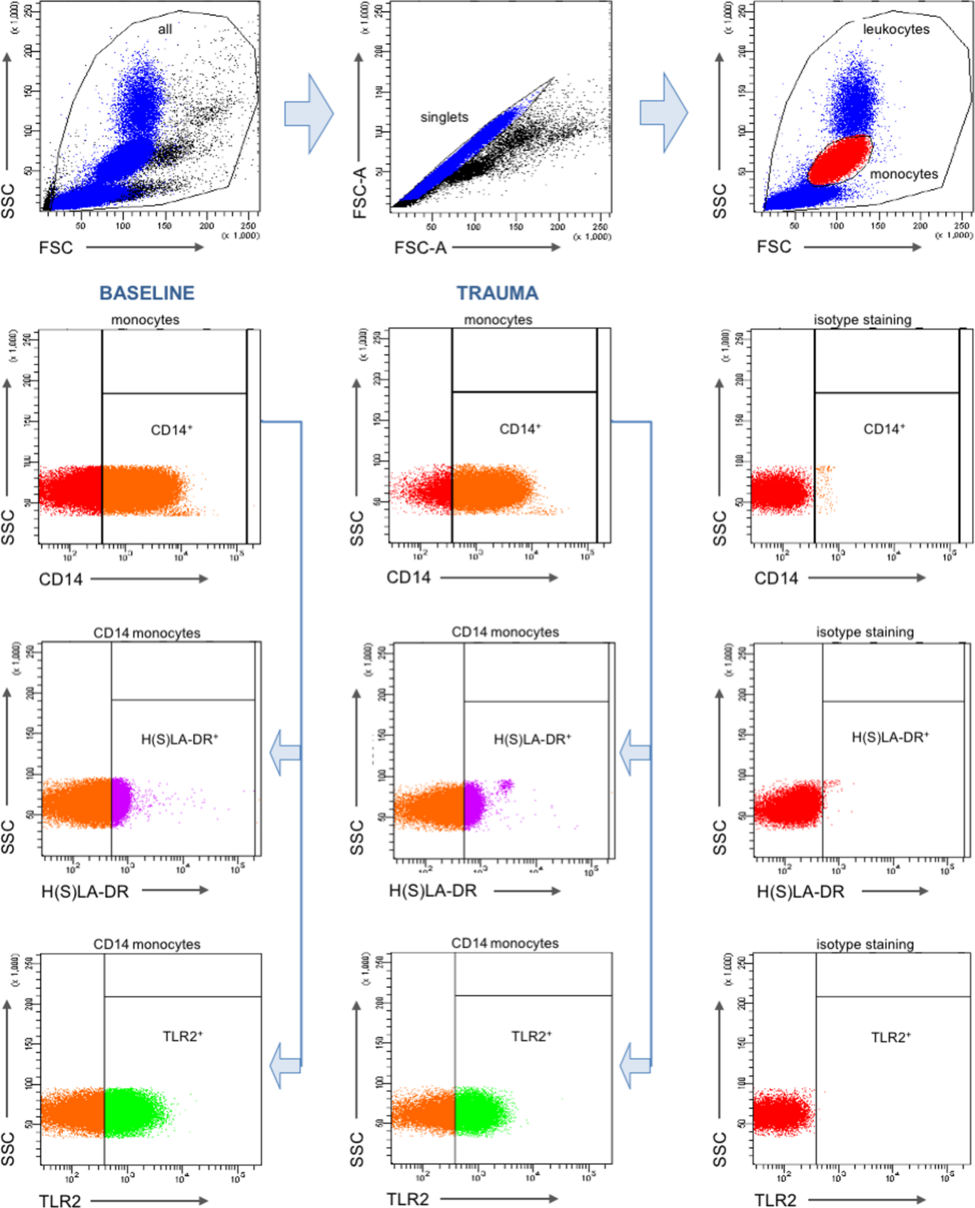
Gating strategy for flow cytometric analyses.

**Fig 2:**
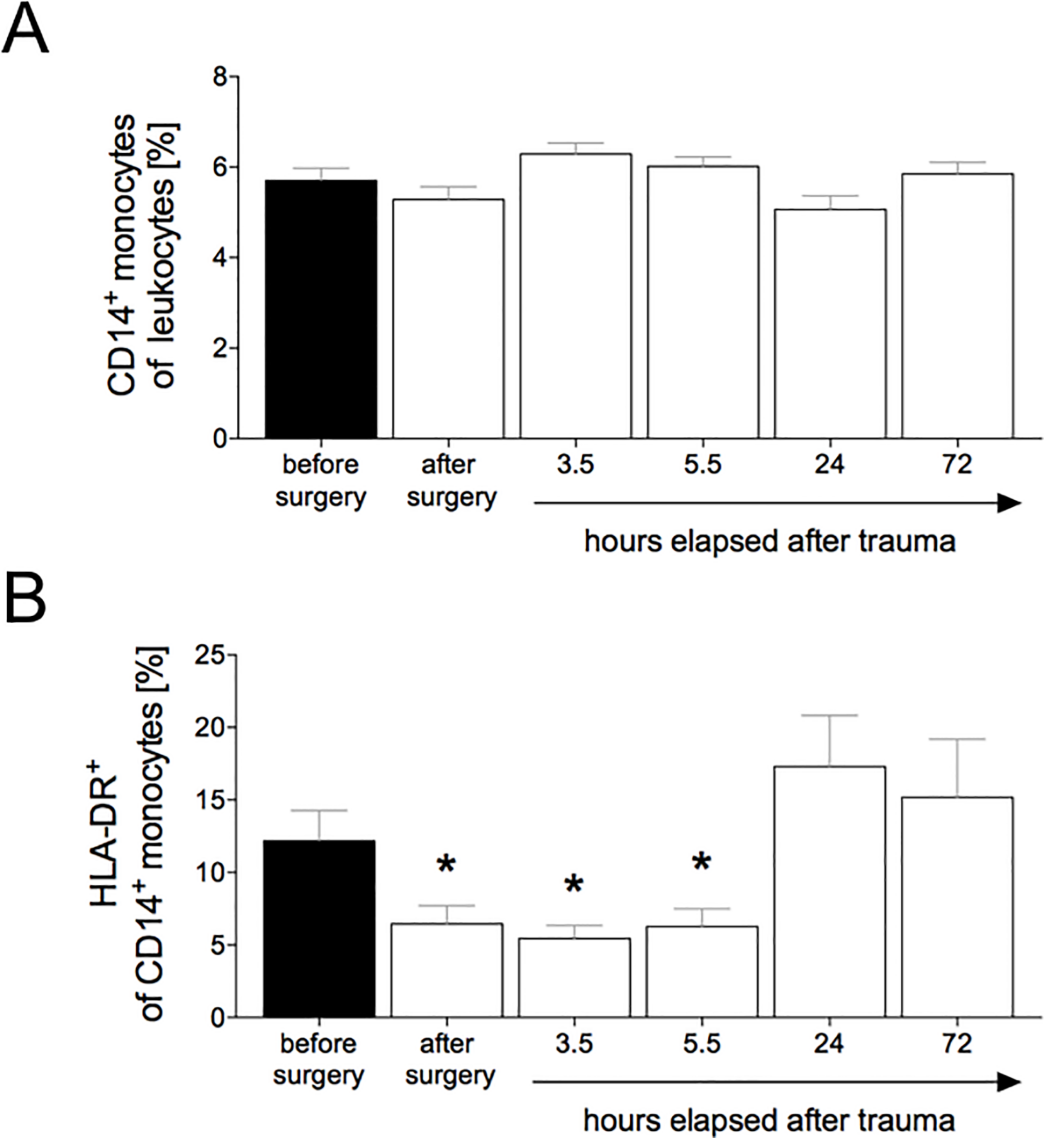
The ratio of porcine peripheral CD14+ monocytes out of total leukocytes was determined in percent. **(A, %)**. Monocytes were characterized by their CD14 surface expression by flow cytometry. The percentage of HLA-DR^+^ expressing porcine peripheral CD14^+^ monocytes of total leukocytes was determined (**B**). Cells were characterized by CD14 and HLA-DR expression by flow cytometry. Blood samples were collected from polytraumatized pigs (n = 12) before trauma as baseline, immediately after surgery/trauma, 3.5 h, 5.5 h, 24 h and 72 h after trauma. Data are shown as mean ± sem. *: p <0.05 *vs*. baseline (before surgery).

Gating strategy is shown in Fig 1. The proportion of peripheral CD14^+^ monocytes out of the total leukocytes was 5.7 ± 0.3% before trauma (Fig 2A). There were no significant changes compared to the initial value over the post-traumatic course of 72h (after surgery: 5.3 ± 0.3%, 3.5 h: 6.3 ± 0.2%, 5.5 h: 6 ± 0.2%, 24 h: 5.1 ± 0.3%, and 72 h: 5.9 ± 0.3%, Fig 2A).

### Surface expression of HLA-DR^+^ on CD14^+^ monocytes

The proportion of peripheral HLA-DR expressing CD14^+^ monocytes out of total leukocytes was initially 11.6 ± 2.2% (Fig 2B). The ratio was significantly lowered directly after surgery compared to baseline (6.5 ± 1.3% *vs*. 11.6 ± 2.2%, p <0.05, Fig 2B). The ratio was also significantly decreased 3.5 h (5.5 ± 0.9%) and 5.5 h (6.3 ± 1.2%) after trauma compared with values obtained before trauma (11.6 ± 2.2%, p <0.05, Fig 2B). 24 h and 72 h post-trauma, the population reached levels comparable to controls (17.3 ± 3.5% and 15.2± 4%, respectively, Fig 2B).

### Surface expression of TLR2 on CD14^+^ monocytes

**Fig 3:**
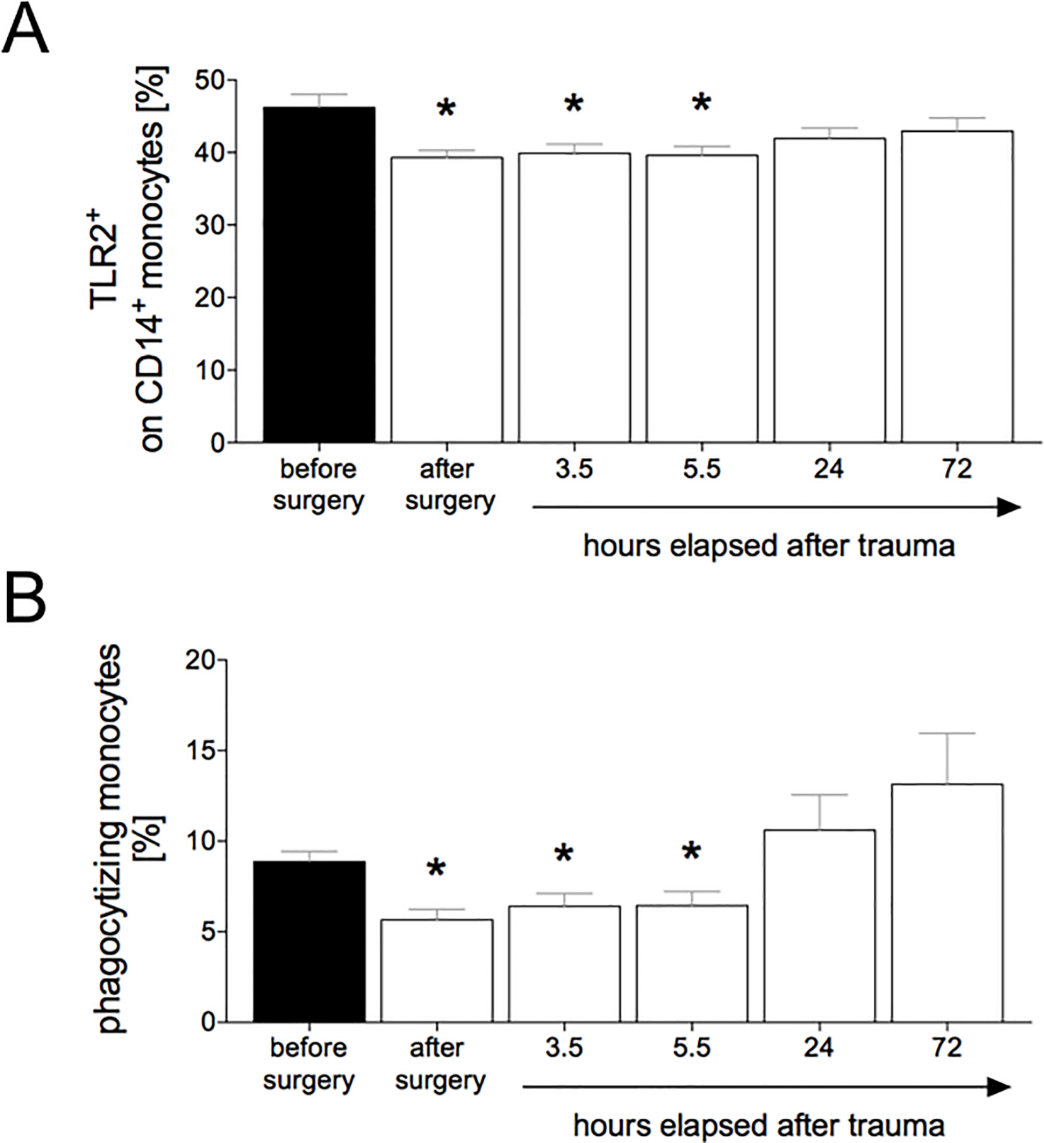
The percentage of TLR2+ expressing porcine peripheral monocytes out of total CD14+ monocytes was determined. (**A**). Cells were characterized by CD14 and TLR2 expression by flow cytometry. Blood samples were collected from polytraumatized pigs (n = 12) before trauma as baseline, immediately after surgery/trauma, 3.5 h, 5.5 h, 24 h and 72 h after trauma. The percentage of phagocytizing porcine peripheral monocytes out of total monocytes was determined (**B**). Whole blood samples were incubated with pHrodo® Red *S*. *aureus* BioParticles® Conjugate. The measurement was performed by flow cytometry. Monocytes were discriminated by forward and sideward scatter. Data are shown as mean ± sem. *: p <0.05 *vs*. baseline (before surgery). Data are shown as mean ± sem. *: p <0.05 *vs*. baseline (before surgery).

Before trauma 45.5 ± 1.8% of CD14^+^ monocytes were expressing TLR2 on their cell surface (Fig 3A). Directly after surgery, the CD14^+^ monocytes expressing TLR2 dropped significantly compared to baseline (39.3 ± 1% *vs*. 45.5 ± 1.8%, p <0.05, Fig 3A). CD14^+^ monocytes expressing TLR2 remained significantly lowered 3.5 h (39.9 ± 1.3%) and 5.5 h (40 ± 1.3%) after trauma compared to values before surgery (p <0.05, Fig 3A). 24 h and 72 h after trauma, CD14^+^ monocytes expressing TLR2s reached levels comparable to control values (42.5 ± 1.5% and 43 ± 1.8%, respectively, Fig 3A).

### Phagocytic activity of monocytes after trauma

8.9 ± 0.6% of monocytes were phagocytizing before trauma (Fig 3B). After surgery, phagocytic activity was significantly decreased compared to baseline (5.7 ± 0.6% *vs*. 8.9 ± 0.6%, p <0.05, Fig 3B). The phagocytizing activity of monocytes remained significantly reduced at 3.5 h (6.4 ± 0.7%) and 5.5 h (6.5 ± 0.8%) after trauma compared to data before trauma (8.89 ± 0.6%, p <0.05, Fig 3B). The phagocytic capability of the monocytes recovered at 24 h and 72 h after trauma and reached values comparable to controls (10.6 ± 2% and 13.1 ± 2.8%, respectively, *vs*. 8.9 ± 0.6%) (Fig 3B).

### Phagocytic activity of monocytes after TRL2 neutralization

**Fig 4:**
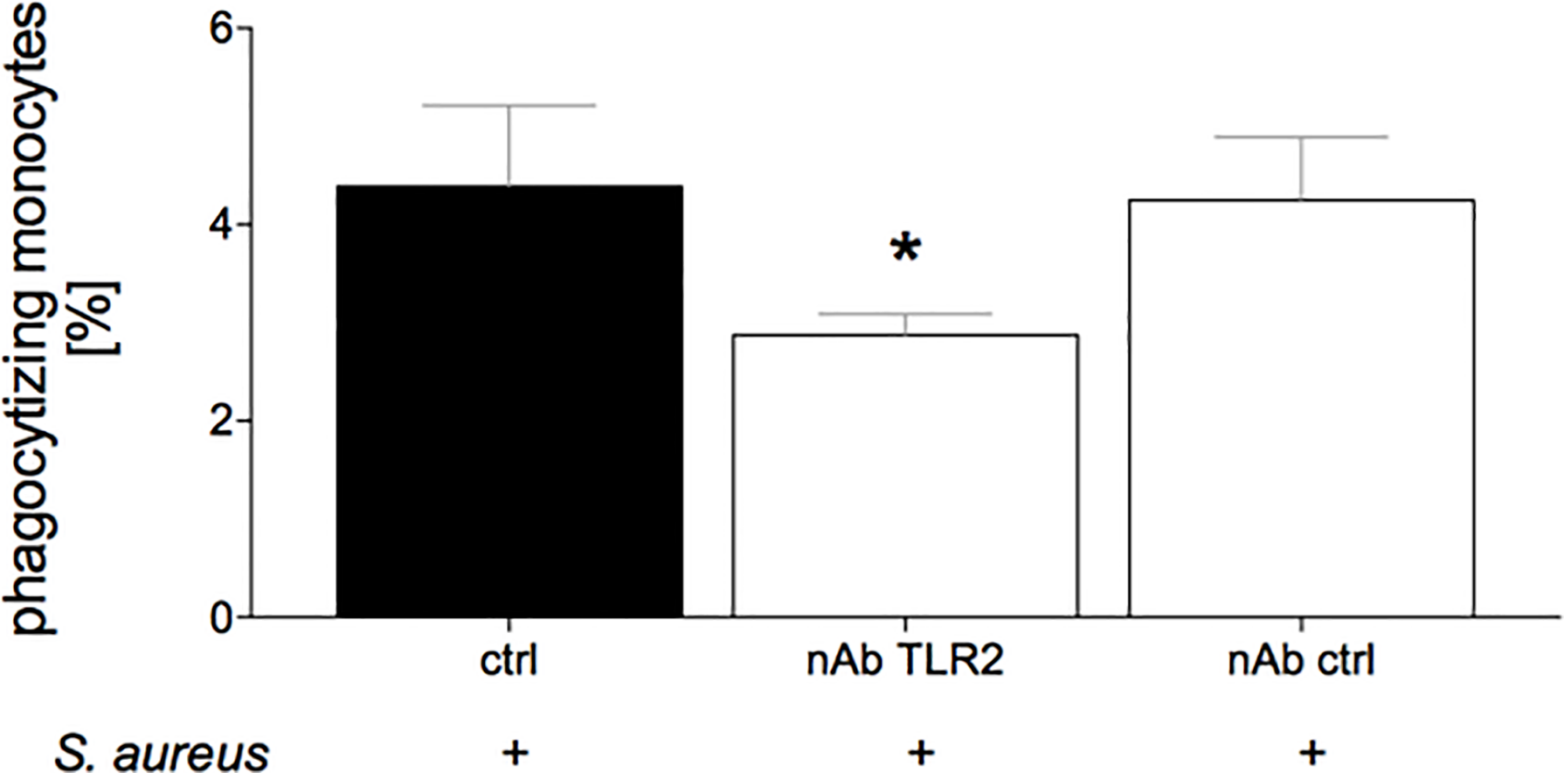
The percentage of phagocytizing porcine peripheral monocytes out of total monocytes was determined. Blood samples were collected from healthy pigs (n = 10). Whole blood samples were incubated in the first step with TLR2 neutralizing (nAB TLR2) or control (nAB ctrl) antibody, or without antibody (ctrl). In the next step, the samples were incubated with pHrodo® Red *S*. *aureus* BioParticles® Conjugate. The measurement was performed by flow cytometry. Monocytes were discriminated by forward and sideward scatter. Data are shown as mean ± sem. *: p <0.05 *vs*. ctrl.

4.4 ± 0.8% of monocytes without prior TLR2 neutralization were phagocytizing *S*. *aureus* bioparticles (ctrl, Fig 4). After TLR2 neutralization (nAB TLR2), the phagocytic activity dropped significantly compared to ctrl (2.9 ± 0.2% *vs*. 4.4 ± 0.8%, p <0.05, Fig 4). The monocytes incubated with the control antibody (nAB ctrl) have shown similar levels of phagocytosis as the ctrl group (4.2 ± 0.6% *vs*. 4.4 ± 0.8%, Fig 4).

## Discussion

Monocytes play a pivotal role in inflammation and show massive functional modulations in trauma patients e.g. in phagocytosis, their maturation and TLR expression or impaired cytokine secretion after *ex vivo* stimulation with endotoxin.[21, 37–39] Several clinical studies and experimental in vivo studies in small animals have shown an altered surface expression of TLR and HLA-DR as well as an impaired phagocytic activity after severe trauma. Despite numerous studies in the last decades, the importance of functional modulations of monocytes after trauma is still not fully understood.

Porcine monocytes as demonstrated by Fairbairn *et al*. (2013) express CD14.[40] Although, Fairbairn *et al*. used another clone of the monoclonal mouse anti pig CD14 antibody (Clone: MIL2), we confirmed (clone: TÜK4) that the porcine model is useful for studying the *in vivo* function of CD14 and monocytes. Furthermore, we could show that the blood level of circulating CD14 positive monocytes does not change significantly over a time course of 72 hours after trauma in a porcine model.

In general, little is known about the expression of H(S)LA, the porcine MHC molecules on monocytes. Chamorro *et al*. (2004) discovered SLA-I, SLA-II-DQ and SLA-II-DR molecules on porcine monocytes, and Raymond *et al*. (2005) observed the expression of MHC-II on porcine monocytes and their increase after treatment with both lipopolysaccharide (LPS) or lipoteichoic acid (LTA) by using flow cytometry.[30, 31] Further analyses have shown that porcine SLA-1*0401 and human leukocyte antigen (HLA) class I HLA-A*0101 can present the same peptides, but in different conformations, demonstrating cross-species epitope presentation.[41] This is so far important, as it has been verified before that human anti-HLA-DR recognizes porcine leukocyte antigens. With regard to the relevance of its expression, Monneret *et al*. (2006) could correlate the persisting lower expression of MHC-II molecules on human monocytes in patients suffering infections who had increased mortality rates, while survivors have shown an increasing MHC-II expression.[42] The authors concluded that the expression of MHC-II may constitute a highly potent marker for the outcome.[42] Regarding trauma, a significantly decreased expression of MHC-II molecules on human monocytes was reported, however, this phenomenon did not correlate with post-injury infections.[10, 29]. In line with those studies, our study shows the post-traumatic maturation of porcine monocytes was investigated for the first time. We could demonstrate a decreased MHC-II expression on porcine monocytes in the early post-traumatic course with a recovery phase after 24 hours. Due to the limited experimental design with regard to the duration of the observational period, we did not evaluate the clinical course, and therefore, we are unable to make a statement regarding the influence of the observed decrease in H(S)LA-DR positive monocytes on the possibly emerging complications in our model. Nonetheless, our findings confirm the data from trauma patients, and indicate at a lower stage of maturation of monocytes with subsequently impaired function after trauma. The relevance of this findings remains to be evaluated in further studies.

The activation of monocytes is triggered *via* TLRs. Thus, despite their maturation as described above, considering functional alterations of monocytes after trauma, it seems reasonable to analyze the TLR expression on monocytes after trauma. The genes of 10 porcine TLRs (TLR1-10) are described and listed in the public nucleotide database. So far, however, little is known about their expression on porcine monocytes and little with regard to porcine severe trauma. Liu *et al*. (2009) demonstrated the gene expression for TLR2, 3, 4, 7, 8 and 9, and have shown a significant upregulation *ex vivo in vitro* for TLR2, 4 and 8, after infection in porcine peripheral blood mononuclear cells.[43] Interestingly, in surgical trauma patients increased TLR2 in comparison to the control group was reported.[44] Lendemans *et al*. (2007), however, have shown a significantly downregulated expression of TLR2 receptor on monocytes within the first 48 hours after severe trauma in patients.[24] This is in line with data reported here, confirming the early decrease in TLR2 expressing monocytes immediately after trauma, indicating that the porcine trauma model represents the changes in TLR2 expressing monocytes, which were observed in trauma patients.

In general, nothing is known about the post-traumatic behavior of porcine monocytes so far and also their phagocytic activity after trauma was investigated for the first time in a porcine trauma model. The expression of TLR2 on CD14^+^ monocytes was decreased simultaneously with the phagocytic activity, in particular directly after surgery, 3.5 and 5.5 hours after trauma. Subsequently, these findings led us to the hypothesis that the impaired TLR2 expression on monocytes is associated with their reduced phagocytic activity of *S*. *aureus* particles in the early post-traumatic course. Previous publications already suggested the dependence of phagocytosis on PRRs and supported our hypothesis. Attempts to correlate the TLR2 expression with the diminished immune activity on monocytes, such as phagocytosis after severe trauma are limited. Sturm *et al*. (2017) have shown a decreased phagocytic activity of human monocytes in the first two days after severe trauma, with a recovery beginning at post-injury day 3.[22] Data from this study confirm the early diminished phagocytosis, which was observed in patients after trauma as well. There is, however, apparently a faster recovery in porcine monocytes as compared to human samples after trauma. Freeman and Grinstein (2014) addressed the association of TLRs to the phagocytic activity before.[20] In brief, TLR activation leads to a so called inside-out activation, increasing the mobility of phagocytic receptors on the cell surface/cell membrane, thereby facilitating the engulfment of particles/pathogens.[20] In another *in vitro* model, it has been demonstrated that specific phagocytosis probably involved recognition of cell wall components that requires participation of a TLR2-dependent pathway.[45, 46] Human peripheral blood monocytes, treated with the TLR2 agonist have shown significantly enhanced phagocytic ability *in vitro*.[47] Consistently, our results have shown as well that early diminished TLR2 expression in porcine monocytes was associated with a simultaneously decreased phagocytizing activity after trauma. To define the role of TLR2 in the phagocytosis response of monocytes against *S*. *aureus*, we investigated the phagocytosis in TLR2-neutralized monocytes obtained from healthy pigs. We could confirm our hypothesis, TLR2 neutralization reduced the phagocytizing rate. Taken together, our data suggest that the decrease of the ratio of TLR2 positive monocytes after trauma may be responsible for the decrease in monocyte phagocytosis, which we have observed in the present study. Combined with results of previous publications, we assume that TLR2 might probably mediate the phagocytizing capability of monocytes for certain bacterial particles in the porcine polytrauma model.

Despite new insights in the porcine cellular physiology, our study has several limitations. First, the very low phagocytizing capacity of the cells may possibly be caused by the use of EDTA and not heparin blood for the assay. Furthermore, it remains to be elucidated, if the early phagocytosis-depression of monocytes will influence the outcome after trauma. Due to the limited observational period (72 hours), our findings cannot be linked to defined outcomes like infections, sepsis, length of stay on ICU or requirement for ventilation which occur way after 72h. Several studies suggest intracellular processes as reason for an impaired post-traumatic function of monocytes. There are different mechanisms of TLR regulation, which have not been addressed here, such as localization and trafficking between the Golgi and the cell surface, interactions of the transmembrane domain or TLR adapter molecules as crucial targets of trauma causing alteration of TLR expression and function.[48, 49] The same applies for the phagocytic activity of monocytes. Seshadri *et al*. observed a consistent phagocytic activity of *S*. *aureus* despite an impaired TLR2 and MHC-II expression.[9] The authors concluded the reduced TLR2 expression can be compensated by other molecules involved in the regulation of phagocytosis. Another limitation of our study is clearly the use of different subspecies of pigs (*German Landrace* vs *Petrain*) in different parts of the study. Additionally to the different subspecies, the “polytrauma-pigs” had to undergo pre-trauma procedures (anesthesia and multiple catheters as described), which might have led to a priming of the immune cells, leading to an increased phagocytic activity before trauma compared to the “healthy” pigs. In future studies, different parts should be executed with specimen of the same subspecies receiving same treatments. However, the results in both parts of our study build on one another and the results and trends are reasonable. Additionally, other populations of monocytes (weakly CD14-positive, CD16-positive cells, etc.) have not been included in the analyses.[50–52]

Our study must be regarded as a first approach to explore such phenomena, if the post-traumatic cellular physiology of pigs is comparable to the one of humans. We started experimentation for monocytes and our findings are promising. However, further studies must be done for different cell lines, even for the other mentioned populations of monocytes in order to truly establish a porcine large animal polytrauma model.

In summary, our results demonstrate that porcine monocytes, undergoing polytrauma, had a decreased TLR2 expression and an impaired maturation as well as a reduced bacterial clearance *via* phagocytosis in the early post-traumatic course. The deranged phagocytizing capacity is highly associated with the reduced TLR2 expression on monocytes, and may provide a new therapeutic target to improving phagocytosis during infection. Moreover, this porcine model is representative for the initially suppressed function of monocytes after trauma in humans.

## Conclusions

- The ratio of porcine monocytes on all leukocytes does not change significantly over the post-traumatic course.
- Porcine monocytes fail in maturation in the early post-traumatic course.
- Porcine monocytes have a decreased expression of TLR2 in the early post-traumatic course.
- Porcine monocytes have an impaired phagocytic activity in the early post-traumatic course.
- Impaired phagocytizing function of monocytes is closely associated with their reduced TLR2 expression.
